# Accelerating Use of Self-measured Blood Pressure Monitoring (SMBP) Through Clinical-Community Care Models

**DOI:** 10.1007/s10900-020-00858-0

**Published:** 2020-06-20

**Authors:** Margaret Meador, Judy Hannan, Debosree Roy, Kate Whelihan, Nana Sasu, Heather Hodge, Joy H. Lewis

**Affiliations:** 1 Clinical Affairs, National Association of Community Health Centers, 7501 Wisconsin Ave., Suite 1100W, Bethesda, MD 20814 USA; 2 Division for Heart Disease and Stroke Prevention, Centers for Disease Control and Prevention, Atlanta, USA; 3 Department of Public Health, School of Osteopathic Medicine in Arizona, A. T. Still University and A.T. Still Research Institute, Mesa, USA; 4 Department of Public Health, School of Osteopathic Medicine in Arizona, A.T. Still University, Mesa, USA; 5 Clinical Affairs, National Association of Community Health Centers, Bethesda, USA; 6 Evidence-based Health Interventions, YMCA of the USA, Chicago, USA

**Keywords:** Self-measured blood pressure monitoring, Home blood pressure monitoring, Collaborative care models, Community Health Center, Hypertension

## Abstract

Self-measured blood pressure monitoring (SMBP), the regular measurement of blood pressure by a patient outside the clinical setting, plus additional support, is a proven, cost-effective but underutilized strategy to improve hypertension outcomes. To accelerate SMBP use, the Centers for Disease Control and Prevention (CDC) funded the National Association of Community Health Centers, the YMCA of the USA, and Association of State and Territorial Health Officials to develop cross-sector care models to offer SMBP to patients with hypertension. The project aimed to increase the use of SMBP through the coordinated action of health department leaders, community organizations and clinical providers. From 1/31/2017 to 6/30/2018, nine health centers in Kentucky, Missouri, and New York partnered with seven local Y associations (local Y) and their local health departments to design and implement care models that adapted existing primary care SMBP practices by leveraging capacities and resources in community and public health organizations. Nine collaborative care models emerged, shaped by available community assets, strategic priorities, and organizational culture. Overall, 1421 patients were recommended for SMBP; of those, 795 completed at least one cycle of SMBP (BP measurements morning and evening for at least three consecutive days). Of those recommended for SMBP, 308 patients were referred to a local Y to receive additional SMBP and healthy lifestyle support. Community and public health organizations can be brought into the health care delivery process and can play valuable roles in supporting patients in SMBP.

## Introduction

Uncontrolled hypertension is one of the leading risk factors for cardiovascular disease. Despite available treatments, only about half of the individuals with hypertension have optimal blood pressure control [1]. Self-measured blood pressure monitoring (SMBP), the regular measurement of blood pressure by a patient outside the clinical setting, along with additional support, is effective in improving control of blood pressure [2], and is part of current hypertension control guidelines [3]. Despite wide international use of SMBP as a standard of care [4–6] as well as the growing national evidence and endorsement of SMBP’s value in diagnosing and managing high blood pressure [2, 3], Ostchega et al. estimated that fewer than 25% of US adults with hypertension engage in any home blood pressure monitoring [7]. The percent of adults with hypertension who engage in SMBP with the recommended frequency and who also work with clinical teams to incorporate readings into their system of care for hypertension is likely much lower. While there are many obstacles to broad adoption of SMBP, there is also keen interest in improving hypertension control and in developing clinical and community partnerships that promote health and well-being. This broadening of the locus of care can help people in their daily efforts to improve their own health [8, 9].

The Centers for Disease Control and Prevention (CDC) worked with three valued health partners from October 2016 to June 2018 to deploy a collaborative approach to attaining improved use of SMBP: The National Association of Community Health Centers (NACHC) representing a clinical arm, the YMCA of the USA (Y-USA) representing a community arm, and the Association of State and Territorial Health Officials, (ASTHO) representing public health.

NACHC is a national advocacy organization for the nearly 1400 community health centers that provide primary care to over 28 million people in America’s most underserved and vulnerable communities. NACHC provides training and technical assistance, conducts research, and develops partnerships to promote and support the work of the health centers. NACHC provided funding and quality improvement coaching to health centers to participate in this project and supported a monthly learning collaborative environment for all partners.

Y-USA is the national resource office supporting 2700 local Y associations (local Ys) to strengthen communities through youth development, healthy living and social responsibility. In 2014, the Y-USA began scaling a 4-month program to support adults with hypertension to develop the habit of monitoring their blood pressure and understand the role of nutrition on their health. Participants received support from local Y staff trained as “Healthy Heart Ambassadors.” In this project, Y-USA helped link local Ys offering this program to health centers implementing SMBP.

ASTHO is the national nonprofit organization representing public health agencies in the United States, with a primary function to track, evaluate, and advise members on health policy and provide them with guidance and technical assistance on improving the nation’s health. In this project, ASTHO worked to connect state and local public health agencies with health center and local Y partners and to define and test the role public health could play in accelerating use of SMBP.

### Aims

This project aimed to increase the use of SMBP through the coordinated action of health department leaders, community organizations and clinical providers. In addition, this project provided an opportunity to develop, document, and evaluate methods to link public health, community, and clinical actions for the promotion and support of SMBP.

## Methods

### Setting and Participants

Relationships were forged at the national level between NACHC, Y-USA, and ASTHO. These national organizations worked together to choose target states, design and launch an innovative SMBP initiative and fund local constituent organizations. From January 2017 to June 2018, nine community health centers in Kentucky, Missouri, and New York worked with seven local Ys and eight local health departments to design, test, and implement collaborative approaches to implementing SMBP.

### Collaborative Approach

There were four main components used to build and implement collaborative SMBP models. These included:

1. Building partnerships between clinical, community, and public health organizations to implement a common definition of SMBP as a tool for hypertension care.
2. Determining SMBP tasks that can be accomplished by a person other than a licensed clinician.
3. Developing collaborative SMBP approaches by localizing best practices and leveraging community and public health resources.
4. Convening a learning community with monthly knowledge sharing opportunities from subject matter experts and peers and utilizing quality improvement coaching for health centers.

#### Partnerships and Common SMBP Definition

The national organizations (CDC, NACHC, Y-USA, and ASTHO) came together to establish project goals and coalesce around a common definition of SMBP. SMBP was defined as a method for individuals with hypertension to take regular measures at home using a home blood pressure monitor sufficient to establish a meaningful pattern of data to manage treatment. A completed SMBP protocol was defined as a patient monitoring their blood pressure at home with at least two measurements a day, morning and evening, for three consecutive days then reporting back to their clinician.

#### SMBP Tasks by Role

The national team inventoried tasks required to support a patient completing an SMBP protocol. Many of these tasks, such as educating patients on using a home blood pressure monitor, subsidizing home blood pressure monitors (e.g., loaning or providing), and training patients on how to communicate blood pressure measurements back to the care team, have been previously identified [10]. Required and optional tasks were detailed.

Tasks were separated by what absolutely must be done by a licensed clinician and those that must be done by the patient. That left tasks that can be accomplished by a non-clinical person—what we will refer to from this point forward as a “SMBP Supporter” (see Table 1: SMBP Tasks by Role).

**Table 1:** Self-measured blood pressure monitoring (SMBP) tasks by role

SMBP tasks by role		
Must be done by licensed clinician	Can be done by SMBP supporter^a^	Must be done by patient
1. Diagnose hypertension 2. Prescribe medication(s) 3. Provide SMBP measurement protocol 4. Interpret patient-generated SMBP Readings 5. Provide medication titration 6. Provide lifestyle modification recommendations	1. Provide guidance on home blood pressure (BP) monitor selection 2. If needed, provide home BP monitor (free or loaned) 3. Provide training on using a home BP monitor 4. Validate home BP monitor against a more robust machine 5. Provide training on capturing and relaying home BP values to care team (e.g., via device memory, patient portal, app, log) 6. Reinforce clinician-directed SMBP measurement protocol 7. Provide outreach support to patients using SMBP 8. Share medication adherence strategies 9. Provide healthy lifestyle education	1. Take SMBP measurements 2. Take medications as prescribed 3. Make recommended lifestyle modifications 4. Convey SMBP measurements to care team 5. Convey side effects to care team

Optional SMBP supporter task	
1. Reinforce training on using a home BP monitor 2. Reinforce training on capturing and relaying home BP values to care team (e.g., via device memory, patient portal, app, log) 3. Reinforce knowledge of behaviors that can trigger high blood pressure	

^a^Medical assistant, community health worker, local public health department/community organization representative, etc

#### Collaborative SMBP Approaches

Local health center/community organization/public health teams determined *how* they could accomplish the tasks detailed by the national team. Local teams assembled tasks into a functional approach or protocol. Teams focused on adapting existing evidence-base primary care practices around SMBP [7] while leveraging unique capacities and resources available through community organizations and public health partners to support their SMBP efforts.

**Fig. 1:**
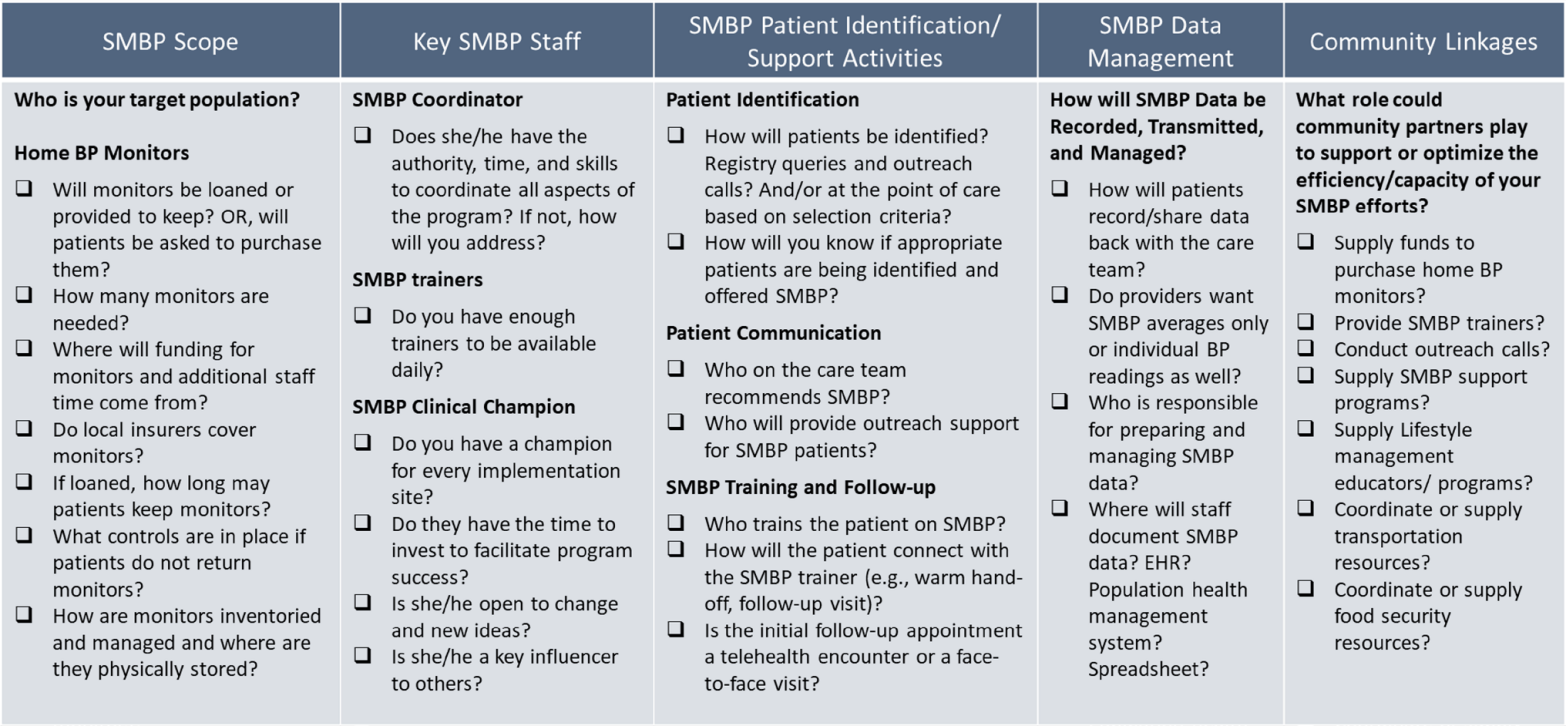
Self-measured blood pressure monitoring (SMBP) model design checklist with key questions

The national team developed the SMBP model design checklist (see Fig. 1: SMBP Model Design Checklist with Key Questions). This checklist is organized into five domains: SMBP scope, key SMBP staff, SMBP patient identification and support activities, SMBP data management, and community linkages. Each domain includes specific questions that need to be answered on the local level. The checklist, along with the detailed tasks and roles were used by the local teams to create clinical protocols and workflows to support hypertension patients using SMBP. When possible, these included public health and community resources.

Teams used quality improvement methods, including workflow and information flow analysis, Plan-Do-Study-Act cycles [11], and annotated run charts to design, test, refine, and implement collaborative SMBP clinical protocols and workflows.

#### Learning Community and QI Coaching

To support health centers and their community and public health partners as they developed their collaborative SMBP approaches, we convened a learning community with monthly knowledge sharing opportunities for subject matter experts and peers. The learning community, which we called our “All Teams Call”, provided a forum to go over key tasks and best practices.

NACHC, Y-USA and ASTHO also held monthly calls with project participants to enable peer to peer learning, capture leading practices, and support program/partnership implementation.

#### SMBP Implementation

Health centers began implementation by identifying adult patients, 18 to 85 years of age who might benefit from SMBP. Health center care teams recommended patients with uncontrolled primary/essential hypertension (defined as a systolic blood pressure ≥ 140 mmHg or a diastolic blood pressure ≥ 90 mmHg) for SMBP based on individual health center protocols, typically through health information technology registry identification and a recommendation or referral from medical providers. From July 2017 to June 2018, identified patients were offered training on SMBP. Patients were given or loaned a monitor and educated on how to use it. The education included proper preparation and positioning to obtain an accurate measurement and how to communicate blood pressure measurements back to the care team. For those using Bluetooth-enabled monitors, patients received training on an associated app that sent measurements to an online portal accessible to their care team. Patients were supported via follow-up phone calls, patient portal messages, and/or text messages.

A summary of clinic and partner characteristics helps set the stage for program implementation. Table 2 provides a profile of these partnering health centers and collaboration partners.

**Table 2:** Collaboration Partners Characteristics

Health center	Patient population served	Health center headquarters location (number of delivery sites)	Urban/rural	Local public health agency	Public health agency service area	Y name
ARcare	62,118	Augusta, AR (39)^b^	Rural	Purchase District Health Department	Ballard County, Carlisle County, Fulton County, Hickman County, and McCracken County	n/a
Shawnee Christian Health Center	3593	Louisville, KY (5)	Urban	Louisville Metro Department of Public Health	Louisville Metropolitan Area, Louisville-Jefferson County	YMCA of Greater Louisville
White House Clinics	29,765	McKee, KY (8)	Rural	Louisville Metro Department of Public Health	Louisville Metropolitan Area, Louisville-Jefferson County	YMCA of Central Kentucky
Finger Lakes Community Health	27,356	Penn Yan, NY (7)	Rural	Yates County Health Department	Yates County	YMCA of Greater Rochester
Hudson River Healthcare	184,404	Peekskill, NY (26)	Urban	Dutchess County Department of Behavioral and Community Health	Dutchess County	YMCA of Rye
Open Door Family Medical Centers	53,896	Ossining, NY (5)	Urban	Westchester County Department of Health	Westchester County	YMCA of Rye^a^
Whitney M. Young, Jr. Health Center	19,499	Albany, NY (2)	Urban	Albany County Department of Health	Albany County	YMCA of Capital District
Affinia Healthcare	43,367	St. Louis, MO (4)	Urban	City of St. Louis Health Department	St. Louis County	Gateway Region YMCA
Samuel M. Rodgers Health Center	23,187	Kansas City, MO (4)	Urban	Kansas City Health Department	Jackson County, Clay County, Platte County and Cass County	YMCA of Greater Kansas City

Clinic characteristic data were extracted from the HRSA’s Health Center Program Uniform Data System (UDS) Resources webpage. Urban/Rural designation of clinic location was based on the 2010 US census bureau definition for urban areas (non-urban areas being designated as rural areas). Public health service area data were obtained from agency webpages and Y population served data were obtained from local organizations, through the Y-USA

^a^The Y worked outside of its service area to support program delivery

^b^Sites in both AR and KY

Some health centers referred all patients recommended for SMBP to community programs and required that they had to agree to use SMBP and also to attend the community program, in order to be counted as an SMBP participant. Other health centers risk stratified their patients, suggesting those who had blood pressure levels up to 160 mmHg systolic or 100 mmHg diastolic utilize community programs to receive lifestyle support, while patients with blood pressure levels ≥ 160 mmHg systolic or ≥ 100 mmHg diastolic received more intensive counseling and education offered by the health center. Once a participant achieved a level of blood pressure < 160 mmHg systolic and < 100 mmHg diastolic, then they were referred to a community program. Patients were identified as having utilized a community program only when they were referred and also agreed to attend. Some health centers had internal staff who could offer lifestyle support services. Patients who were offered lifestyle support programs at their health center were not referred to a community program.

### Data Collection and Analysis

#### Qualitative Data

Qualitative data were obtained via a face-to-face meeting of all participating organizations and through semi-structured interviews.

The face-to-face meeting took place in March 2018, where health center, Y, and public health teams presented and described their SMBP processes from start to finish. These presentations were standardized by a PowerPoint template that included partners, SMBP patient selection criteria, community referral criteria, patient identification process, patient recommendation/enrollment/training approach and SMBP data management. These also included a reflection about what is unique, innovative, or powerful about each team’s approach, recommendations and lessons learned, and workflow diagrams. Collaborative approaches to implementing SMBP were summarized to understand the varying collaborative models and to detail tasks completed by non-clinical partners.

Key informant semi-structured interviews with each health center project lead occurred in May and June 2018. These covered the nature of their community/public health partnerships, how these partners were integrated into SMBP approaches, sustainability plans, and lessons learned. Each interview was audio recorded and transcripts were entered into NVIVO 12 Plus [12]. We identified specific questions from the interview guide and their associated answers prior to analysis and coded for thematic inferences.

#### Quantitative Data

Monthly reports aggregated at the health center level included number of patients recommended for SMBP, referral to a community program, and patient use of SMBP. These data were collected via a combination of electronic health records (EHR) or population health management system documentation and manual tracking. Most health centers did not have standard places in the vital signs section of their EHR for out-of-office blood pressure measurements or other standard places to document SMBP-related data elements. Five health centers customized their health information technology (HIT) systems to allow for the entry of out-of-office measurement and related SMBP data elements to their systems and were able to report SMBP measures electronically; others tracked out-of-office blood pressure measurements and SMBP-related data using Microsoft (MS) Excel spreadsheets.

This study was determined to be exempt by the AT Still University – Arizona Institutional Review Board (IRB) from continuing IRB review. Interviews with key informants were performed with verbal consent, aggregated, and anonymized, and written consent was not collected. A waiver of written informed consent was granted based on 45 CFR 46.116(d): “Obtaining written consent would be the only way in which interviewees could be linked to their responses.”

## Results

The full project timeline was 18 months (January 2017–July 2018); however, the implementation and execution of the SMBP models largely reflects a 7–11-month timeframe. Health centers and their collaborative partners’ implementation dates ranged between August 2017 to January 2018, depending on the time needed up front to develop their collaborative model, obtain home blood pressure monitors, prepare staff, configure HIT systems, etc.

### Qualitative Results

#### SMBP Tasks and Collaborative Approaches Results

All nine health centers developed different approaches to implementing SMBP. Table 3 illustrates the various SMBP-related tasks in emerging collaborative approaches that were accomplished by health centers, community organizations, or public health agencies in this project. Eight of the nine health centers established collaborative approaches where external organizations assisted with tasks we identified that could be performed by an SMBP Supporter.

**Table 3:**
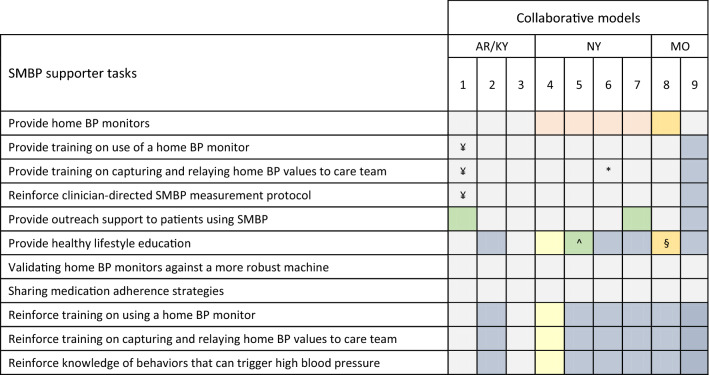
Self-measured blood pressure monitoring (SMBP) collaborative models

¥Some assistance by pharmacy students

*Some assistance by Americorps volunteers

^Both local health Dept. and local YMCA

§Both American Heart Association and YMCA
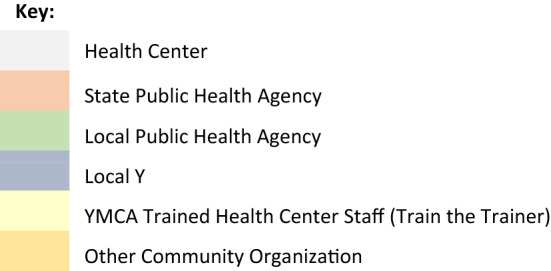

Community and public health organizations assisted most with: providing home blood pressure monitors; providing healthy lifestyle education; reinforcing training on use of a monitor; reinforcing training on capturing and relaying home BP values and reinforcing knowledge of behaviors that can trigger high blood pressure.

In New York, all four participating health centers worked with the New York State Department of Health, which purchased home blood pressure monitors so they could be loaned to patients for SMBP. At the local level, three New York health centers referred patients out to a local Y program, and the fourth center had their health center staff become certified as Y Healthy Heart Ambassadors to deliver the Y program at their health center. One New York health center also partnered with their local public health agency to conduct outreach calls and home visits to patients to support their SMBP use after they had taken a monitor home, while another worked with their local public health department to obtain educational resources that would support their SMBP efforts. Finally, one of the New York health centers worked with Americorps [13] volunteers to assist their care teams with SMBP training and data management.

In Kentucky, one site partnered with a local university to have pharmacy students train patients on SMBP inside the health center, while another site within this same health center organization partnered with a local public health department, which completed outreach phone calls to patients who had taken home a home blood pressure monitor.

In Missouri, one health center established regular office hours (2 days per week) for local Y staff to deliver the local Y program on location at their health center. The local Y staff were woven into their SMBP model to provide initial training on SMBP to patients as well as SMBP reinforcement and healthy lifestyle education. A second Missouri health center collaborated with multiple partners, including the American Heart Association, who assisted with purchasing home blood pressure monitors and delivering healthy lifestyle education at the health center, as well as the local Y, to which the health center also referred patients.

**Fig. 2:**
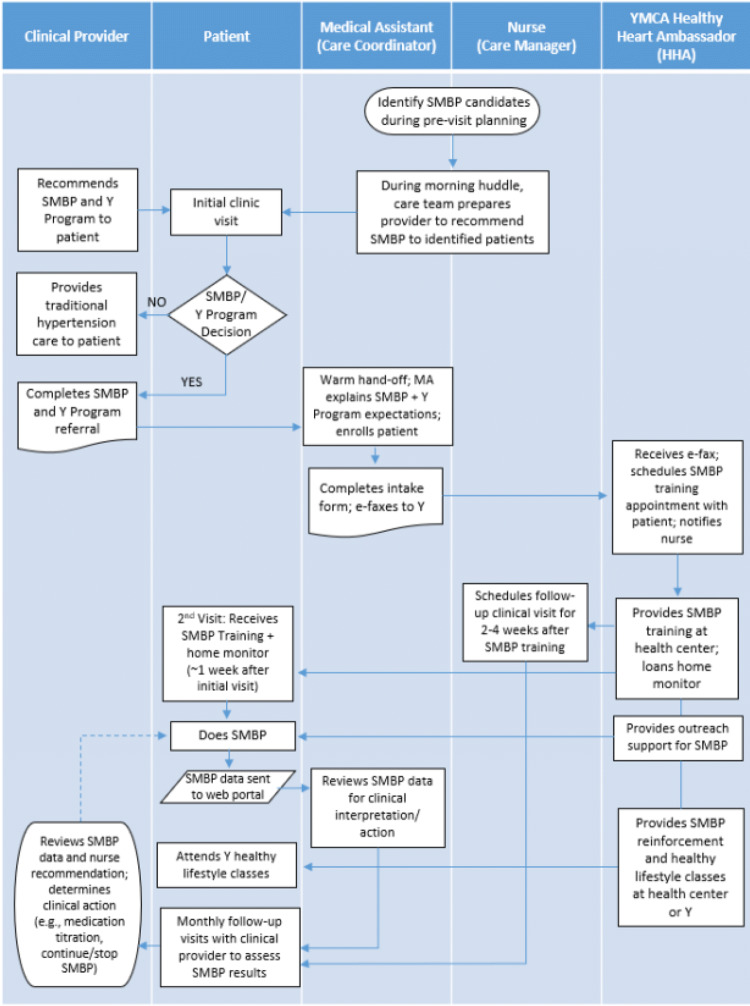
Example—collaborative self-measured blood pressure monitoring (SMBP) workflow

Most health centers also sought other kinds of collaboration as part of their overall SMBP approach not indicated in Table 3 to help enhance supports or address barriers around hypertension control (e.g., additional lifestyle education or exercise opportunities, transportation services, fresh food pantries, etc.). Figure 2 depicts an example of a workflow that one health center created with its local Y partner detailing various roles and patient flow designed to accomplish all the necessary SMBP tasks.

#### Key Informant Interviews

Key informant interviews of health center staff sought to reveal whether clinical-community collaborations helped support SMBP adoption and what elements were important for successful collaborations. The primary theme that emerged was that collaborations were beneficial where a “win–win” situation could be created for both the health center and community-based organization (CBO). Some health centers forged new partnerships after assessing their current SMBP needs and sharing this information with their local health department, who then helped the health center consider available capabilities offered by CBOs equally eager to discover new clientele to whom they might offer beneficial services.

Three sub-themes surfaced: (1) learning about CBO assets relevant to supporting SMBP, (2) establishing trust, and (3) developing an empowered working relationship between clinical and CBO staff. Three health center interviewees shared that learning about available community resources was a critical first step in successful collaboration. Others commented on the importance of being able to trust the CBO. For partnerships with local Ys, trust came from knowing the local Ys use certifications and tested training curricula. “With their Healthy Heart Ambassadors, I know that once those people get selected or hired, they are put through a training… They actually have to go through some sort of certification…”.

Health centers that developed new or expanded upon existing partnerships often found that staff from CBOs and/or local public health departments could operate as a part of the expanded care team, sharing information bi-directionally (and confidentially) about SMBP patients. “All…sets of staff…were coordinating with all the different patients but the burden of who was tracking was reduced from the collaboration.” It was important that partners empowered each other to do the work they were tasked with while providing the space and opportunity to function in the best interest of the patient. This empowerment distributed the burden of supporting SMBP and enabled partnerships to provide sustainable healthy lifestyle support and address barriers to SMBP related to costs, transportation, and access to home blood pressure monitors.

### Learning Community and QI Coaching Results

We synthesized and collated information and learning into an SMBP Implementation Guide [14]. This implementation guide is designed to help health care delivery organizations implement SMBP into practice or optimize their existing SMBP processes. From this process, we developed several SMBP-related videos, including two that showcase various SMBP models with clinical and community partners: (1) *Self*-*measurement: How Patients and Care Teams are Bringing Blood Pressure to Control* and (2) *Taking Control of My Blood Pressure: Natalia’s Story*, which shares the story of a patient whose blood pressure was successfully controlled through the support of a collaborative SMBP model in Missouri. The videos can be found at http://www.nachc.org/taking-control-of-my-blood-pressure-patient-stories/.

These videos provide testimony to the impact that SMBP had on the patients’ engagement and efficacy in controlling their BP. In addition, these videos emphasize the following project findings:

- SMBP training, SMBP outreach assistance/reinforcement, healthy lifestyle support, and securing home blood pressure monitors are great activities for which community and public health organizations can play a valuable role.
- SMBP is a departure from the outdated practice of office blood pressure reading as the gold standard; this paradigm shift requires change management.
- Clinician buy-in is essential; one key component is for them to understand how the investment in SMBP will help their hypertension patients get to control faster and become more engaged in their care.
- Community partnerships take time, dedicated staff at both organizations, and clear communication.

#### Quantitative Results

**Fig. 3:**
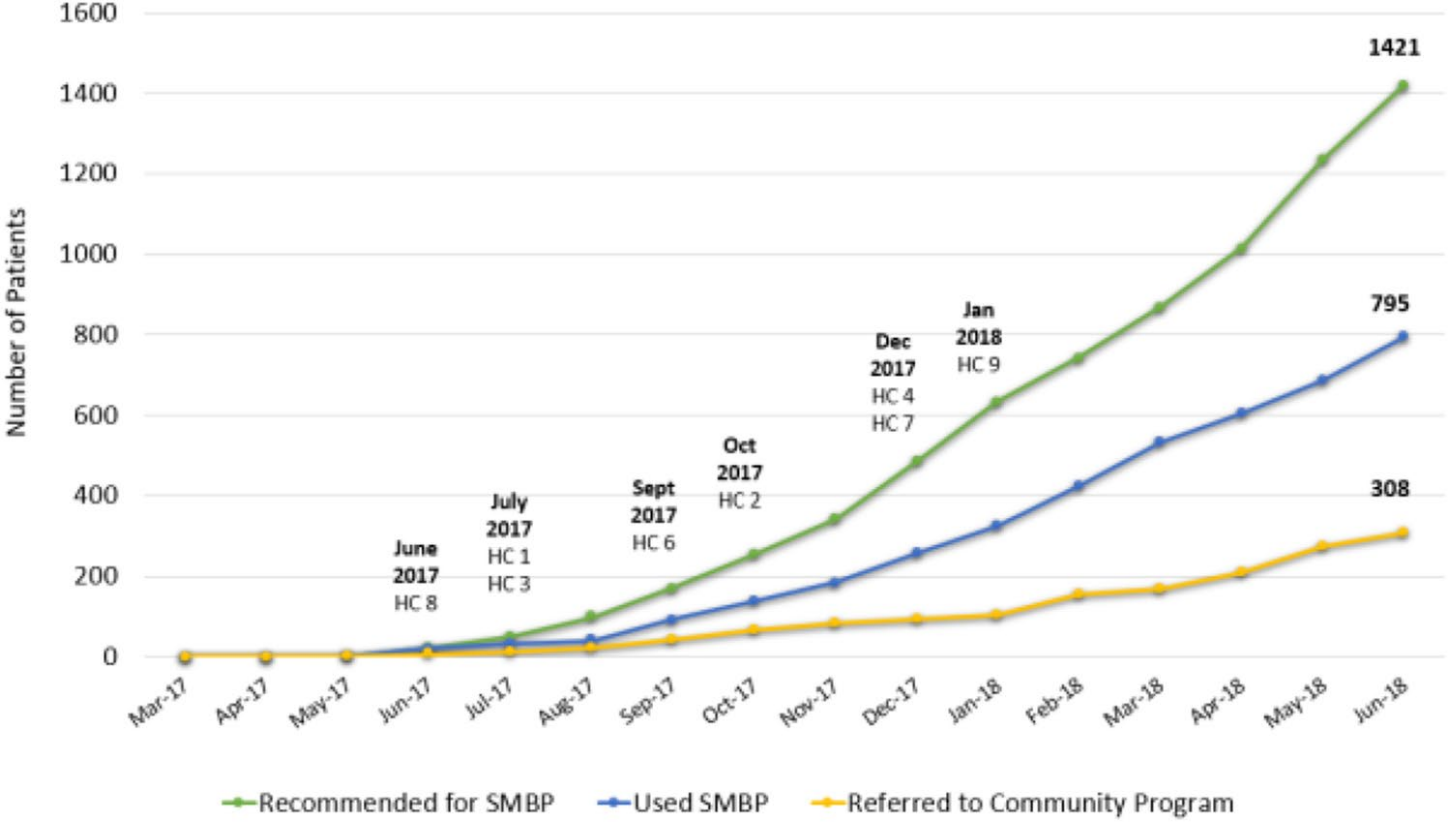
Overall self-measured blood pressure monitoring (SMBP) recommendation and use and community referral results with health center implementation date

By the project’s conclusion the nine participating health centers reported that 1421 patients with uncontrolled hypertension received a recommendation or referral to SMBP. Of those, 795 successfully completed at least one SMBP protocol (SMBP use). In addition, 308 of the 1421 patients received referrals for additional community program support. Health center implementation dates varied between June 2017 and January 2018 (Fig. 3).

## Discussion

Improving hypertension control in this nation is of vital importance to reducing heart attacks and strokes and will likely take the collective actions of many. Effectively using SMBP to diagnose and manage hypertension and engage patients in the control of their hypertension is a key but not necessarily easy task. Collaborative SMBP approaches reveal ways health care delivery organizations may partner with community organizations and public health agencies to expand their available resources, both to mitigate capacity barriers and increase support to patients using SMBP, such as providing healthy lifestyle education programs. Moreover, SMBP may serve as an entry activity to connect clinic activities and community supports more broadly.

By bringing in community partners and assessing which SMBP tasks could be accomplished by someone other than a clinician, we encouraged additional models for SMBP programs. Clinics utilized members of the expanded care team, like medical assistants or community health workers, and went outside of the health center to leverage resources in the local public health department or in community organizations, like the Y.

There was impressive variation in how collaborating organizations chose to accomplish SMBP support tasks. All nine health centers developed different SMBP care models. This variation was shaped by strategic priorities, organizational assets and culture, and available community resources. For example, most health centers that had a Y program in proximity capitalized on the assets and resources the Ys had to provide reinforcement for SMBP and lifestyle education. Those without a local Y program partnered with public health agencies, pharmacy schools, food pantries, transportation services, and other non-profit health organizations like the American Heart Association. The SMBP Collaborative Models table illustrates the variation in how health centers integrated community and public health partners into a seamless SMBP model and demonstrate the ability to utilize partnerships to support patients as they adopt SMBP.

Primary care organizations and providers might be concerned about taking on SMBP because of the number of tasks involved and the current poor reimbursement for devices and support related activities. In 2017, Medicare unbundled CPT code 99091, which allows providers to be reimbursed for time spent on collection and interpretation of health data that is generated by a patient remotely, digitally stored, and transmitted to the provider. Two additional CPT codes have recently been developed that offer pathways for reimbursement for activities related to SMBP and were released in January 2020 [15].

Integrating SMBP into routine hypertension care is important and collaborative partnerships between care delivery organizations, public health agencies, and community organizations may make this practice more sustainable. Further, collaborative care models to support SMBP may be an important step in developing medical and community partnerships that promote health and well-being and broaden the locus of care to help people in their daily efforts to improve their own health.

This paper offers a recipe for health systems to implement SMBP now, despite identified barriers, and in a way that may more fully engage participants.

### Limitations

This project was undertaken in three states where Ys with hypertension support programs already existed. The findings from the collaborations in these states will not necessarily be generalizable to other communities. Because we saw so many different collaborative approaches to SMBP emerge, there was variation both in the timeframe and the way in which SMBP was implemented. This lack of uniformity also limits the generalizability of the findings. However, we believe the variety of approaches and the creativity observed can inform other health centers and communities as they try to implement SMBP programs.

Similarly, the methods by which health centers gathered and reported data for the monthly reports varied. The data we report for the number of people recommended for SMBP, those referred to a community program, and those who used SMBP were based on monthly aggregates provided by each health center. For some centers, these were documented manually using MS Excel spreadsheets, as EHRs currently do not have locations for recording this type of patient-generated information as part of their standard configuration. These data are thus not part of an official medical record and as they are de-identified, the individual participation cannot be verified. This points to a difficulty with implementing home-based monitoring programs in an electronic health record world. We feel comfortable that the reports provided to us reflect the best attempts at accuracy by all centers and that those health centers that developed customized HIT systems to support SMBP were able to provide accurate referral and SMBP use. Further, this paper does not delve into patient experiences around SMBP or their blood pressure control outcomes compared to hypertension patients receiving traditional care; however, future work will examine both areas. While our results are not generalizable, the breadth of SMBP models that emerged provides a menu of approaches that may be identified as applicable to a range of unique clinical-community situations.

### Future Directions

Given the evidence for SMBP in hypertension diagnosis and management [6], national efforts are needed to increase clinical uptake and patient demand for SMBP. Improving reimbursement for home blood pressure monitors and SMBP activities (e.g., training patients, SMBP data management, etc.) would decrease cost barriers. Facilitating use of patient-generated data, including technology that integrates SMBP measurements into EHRs/HIT systems is also an opportunity to increase SMBP use. Most EHRs do not have standard places in which SMBP measurements can be documented in discrete data fields and presented effectively to inform clinical decision-making.

The disinclination among EHR and other HIT vendors to build SMBP data fields and related forms or templates into their systems may stem from the fact that out-of-office measurements are currently not fully accepted in clinical quality measures, even though they have been shown to be more accurate than a single office measurement [5]. Assuring that out of office measurements have an appropriate place in clinical quality measures may be the most important next step to influence reimbursement and technology supporting SMBP.

## Conclusion

Following current guidelines for the diagnosis and management of hypertension includes appropriate use of self-measured blood pressure [5]. This practice is not close to being universal. We imagine a world where people are diagnosed readily, using home monitors, and where all patients with hypertension have a monitor and are trained on how to use the monitor. We can’t imagine a world where people don’t have access to taking their own temperature, weighing themselves, or using a glucose monitor to assist in the diabetic care. Given the toll that hypertension takes on our nation, and our current performance of only about 50% of people with hypertension having it controlled, there is a compelling role for increased support for the use of home monitoring.

Our experience confirms that clinical and community partners can design unique programs where patients can successfully incorporate SMBP into their care regimen. These programs can vary considerably and can utilize extensive partnerships where non-clinical SMBP supporters can play key roles in promoting and expanding SMBP.

## References

[1] Wall HK, Ritchey MD, Gillespie C, Omura JD, Jamal A, George MG (2018). Vital Signs: Prevalence of key cardiovascular disease risk factors for Million Hearts 2022 — United States, 2011–2016. Morbidity and Mortality Weekly Report.

[2] Community Preventive Services Task Force. (June 2015). The Guide to Community Preventive Services: Cardiovascular Disease: Self-Measured Blood Pressure Monitoring Interventions for Improved Blood Pressure Control – When Combined with Additional Support. Retrieved from https://www.thecommunityguide.org/findings/cardiovascular-disease-self-measured-blood-pressure-with-additional-support.

[3] Whelton PK, Carey RM, Aronow WS (2018). 2017 ACC/AHA/AAPA/ABC/ACPM/AGS/APhA/ASH/ASPC/NMA/PCNA Guideline for the Prevention, Detection, Evaluation, and Management of High Blood Pressure in Adults: A Report of the American College of Cardiology/American Heart Association Task Force on Clinical Practice Guidelines. Journal of the American College of Cardiology.

[4] Parati G, Stergiou GS, Asmar R (2010). European Society of Hypertension Practice Guidelines for home blood pressure Monitoring. Journal of Human Hypertension.

[5] Nerenberg KA, Zarnke KB, Leung AA (2018). Hypertension Canada’s 2018 Guidelines for Diagnosis, risk assessment, prevention, and treatment of hypertension in adults and children. Canadian Journal of Cardioliogy.

[6] The Japanese Society of Hypertension (2014). Chapter 2. Measurement and clinical evaluation of blood pressure. Hypertension Research.

[7] Ostchega Y, Zhang G, Kit BK, Nwankwo T (2017). Factors associated with home blood pressure monitoring among U.S. adults: National Health and Nutrition Examination Survey, 2011–2014. American Journal of Hypertension.

[8] Sanders J, Solberg B, Gauger M (2013). Breaking barriers to care: A community of solution for chronic disease management. Journal of the American Board of Family Medicine.

[9] Casey BR (2013). Innovations in primary prevention: Emerging research from CDC’s Prevention Research Centers. Journal of Primary Prevention.

[10] Centers for Disease Control and Prevention. (2014). Self-Measured Blood Pressure Monitoring: Actions Steps for Clinicians. Atlanta, GA: Centers for Disease Control and Prevention, US Dept of Health and Human Services.

[11] Moen R.D. & Normal C.L. (2010). Circling back – clearing up myths about the Deming cycle and seeing how it keeps evolving. Quality Progress, 22–28. Retrieved from http://www.apiweb.org/circling-back.pdf.

[12] QSR International Pty. Ltd. Nvivo 12 Plus. Retrieved from https://www.qsrinternational.com/nvivo/nvivo-products/nvivo-12-plus.

[13] Corporation for National and Community Service. AmeriCorps. Retrieved from https://www.nationalservice.gov/programs/americorps.

[14] National Association of Community Health Centers. (2018). Self-measured Blood Pressure Monitoring: Implementation Guide for Health Care Delivery Organizations. Retrieved from https://www.nachc.org/wp-content/uploads/2018/09/NACHC-Health-Care-Delivery-SMBP-Implementation-Guide-08222018.pdf.

[15] Centers for Medicare & Medicaid Services. (2019). Medicare Program: CY 2020 Revisions to Payment Policies under the Physician Fee Schedule and Other Changes to Part B Payment Policies; Medicare Shared Savings Program Requirements; Medicaid Promoting Interoperability Program Requirements for Eligible Professionals; etc. Retrieved from https://www.federalregister.gov/d/2019-24086.

